# Healthcare Innovations to Address the Challenges of the COVID-19 Pandemic

**DOI:** 10.1109/JBHI.2022.3144941

**Published:** 2022-01-25

**Authors:** Metin Akay, Shankar Subramaniam, Colin Brennan, Paolo Bonato, Charlotte Mae K. Waits, Bruce C. Wheeler, Dimitrios I. Fotiadis

**Affiliations:** Department of Biomedical EngineeringUniversity of Houston 14743 Houston TX 77204 USA; Department of BioengineeringUniversity of California at San Diego 548652 La Jolla CA 92093 USA; Kibur Medical, Inc. Boston MA 02116 USA; Department of Physical Medicine and Re habilitationHarvard Medical School 1811 Boston MA 02115 USA; Department of Biomedical EngineeringUniversity of Houston 14743 Houston TX 77204 USA; Department of BioengineeringUniversity of California at San Diego 548652 La Jolla CA 92093 USA; Department of Biomedical Research, Institute of Molecular Biology and BiotechnologyFORTH 54578 Ioannina Greece; Department of Materials Science and Engineering, Unit of Medical Technology and Intelligent Information SystemsUniversity of Ioannina 37796 45110 Ioannina Greece

**Keywords:** COVID-19, healthcare innovations, public healthcare

## Abstract

We have been faced with an unprecedented challenge in combating the COVID-19/SARS-CoV2 outbreak that is threatening the fabric of our civilization, causing catastrophic human losses and a tremendous economic burden globally. During this difficult time, there has been an urgent need for biomedical engineers, clinicians, and healthcare industry leaders to work together to develop novel diagnostics and treatments to fight the pandemic including the development of portable, rapidly deployable, and affordable diagnostic testing kits, personal protective equipment, mechanical ventilators, vaccines, and data analysis and modeling tools. In this position paper, we address the urgent need to bring these inventions into clinical practices. This paper highlights and summarizes the discussions and new technologies in COVID-19 healthcare, screening, tracing, and treatment-related presentations made at the IEEE EMBS Public Forum on COVID-19. The paper also provides recent studies, statistics and data and new perspectives on ongoing and future challenges pertaining to the COVID-19 pandemic.

## I. Introduction

The severe acute respiratory syndrome coronavirus 2 (SARS-CoV-2), and the associated coronavirus disease 2019 (COVID-19) have greatly impacted our lives. This pandemic has revealed how our healthcare system was not prepared for this major challenge, especially with respect to treatment, rapid diagnosis and tracking, as well as limited hospital equipment, staff members, and resources. COVID-19 may become one of the deadliest pandemics of the 21^st^ century and continues to strain resources available not only to healthcare workers, but also the general public, despite the increasing number of individuals vaccinated against the virus.

## II. Procedures

The Institute of Electrical and Electronics Engineers (IEEE) Engineering in Medicine and Biology Society (EMBS) convened an international COVID-19 forum on November 12–15, 2020 and invited twenty-nine scientific researchers and innovators, industry leaders, clinical experts, and policy makers from the USA, Europe, and China to participate as plenary speakers and to provide a comprehensive and multi-dimensional perspective on the pandemic crisis. The forum had four symposiums to discuss the challenges in healthcare, screening, tracing, and treatment, with 2012 attendees participating in total. The forum featured plenary talks from clinicians on the front lines of pandemic care, industry experts, entrepreneurs, and academic researchers from leading universities and policy makers. These talks were followed by a question and answer (Q&A) session led by the chairs of the organizing committee; both the plenary talks and the panel discussed current and future challenges in combatting the COVID-19 pandemic. We summarize in this white paper those presentations including panel discussions, data presented, and comments made during the sessions. Consequently, this paper could serve as a comprehensive source of information on the healthcare innovations and strategies to address the COVID-19 pandemic. Accordingly, we identified four major grand challenges defining the problems we need to solve now and over the long-term: Healthcare, Screening, Tracing and Treatment.

## III. Grand Challenges in COVID-19 Healthcare

The COVID-19 pandemic has broadly impacted all segments of health care and society since the first reports of viral outbreak in Wuhan, China in December 2019.The global effort to contain and mitigate the virus has been modestly successful with the availability and world-wide deployment of multiple COVID-19 vaccines. The COVID-19 pandemic has exposed the fragility of national healthcare systems; as a global biomedical engineering community, we believe this is an opportunity to contribute our experience and expertise to solve the grand challenges that collectively face us. We invited a broad spectrum of experts in each of these areas to inform us of the details of each of these grand challenges. They informed us of the unexpected early indications of both the acute and the chronic negative impacts of the viral infection on patient respiratory, cardiac and nervous systems and resulting co-morbidities.

Similar to other diseases, several factors and comorbidities influence patient outcomes. For example, an individual's socioeconomic and education status highly impacts not only mortality, but also the length of hospital admission. Males, the aged, ethnic and racial minorities, and those with co-morbidities (e.g., diabetes, congestive heart failure, obesity) were at a higher risk of worse clinical outcomes [1]–[3]. However, as Dr. Ross Zafonte mentioned during his talk, 47% of hospitalized COVID-19 patients had no comorbidities, suggesting this virus can impact not only vulnerable populations, but also otherwise healthy individuals [1]. Early reports estimated the mortality rate of individuals hospitalized with COVID-19 to be >30% [1], [2]. New data from South Africa examined the mortality rate of patients hospitalized during each wave of coronavirus infections. They found the recent wave, which began in November 2021 had a 2.7% mortality rate compared to ∼20% mortality rate. This lower mortality rate is generally attributed to the availability of vaccines as well as the omicron variant, whereas other variants (e.g., delta) were more prevalent in earlier waves where there were no vaccine options available [4], [5].

Further, mental health issues were reported in recovered patients and in the general population due to the social isolation requirements imposed by public health authorities. Novel methods under development or already deployed for rapid and early detection of infection were described in addition to ways of tracing contacts between healthy and infected individuals using mobile phone technology to minimize and prevent spread of the COVID-19 virus. We were informed of new vaccine technologies that could further decrease the development time and make for more potent anti-virals and about other approaches to treat patients, such as neutralizing anti-COVID 19 antibodies, to expanding treatment options and complement vaccines.

### A. COVID-19 Effects on the Respiratory System

Several speakers discussed the effect of SARS-CoV-2 on the respiratory system, specifically mentioning how the virus targets the angiotensin-converting enzyme 2 (ACE2) receptor to enter cells [3], [6]–[12]. This receptor is commonly found on lung epithelium, but is also present in other tissues, namely, the gastro-intestinal tract and cardiovascular system. Due to the high concentration of ACE2 receptors within the lung epithelium and primary transmission of the virus through respiratory droplets, the lungs are largely affected during the early stages of COVID-19. Hallmark symptoms of COVID-19 include fever, cough, rhinorrhea, and labored breathing (i.e., dyspnea) [2], [8], [12], [13]. These can ultimately lead to pneumonia or acute lung injury, which often requires intensive care and oxygen therapy [8]. On chest computed tomography (CT) scans, COVID-19 patients present with bilateral ground-glass opacities and diffuse alveolar damage in all lobes, which correlated with acute respiratory distress syndrome (ARDS) [2], [6], [8], [14].

### B. COVID-19 Effects on the Cardiovascular System

Unlike previous coronaviruses (e.g., SARS-CoV-1 and MERS-CoV), multiple studies have identified cardiac involvement during an active infection of SARS-CoV-2 as well as prolonged effects after recovery. Myocarditis has been a common finding among several studies and can include edema, hyperemia, or fibrosis/scarring of the myocardium and includes general inflammation [7], [15]. Levels of troponin T (TnT) is commonly associated with myocardial injury and was found to be associated with increased mortality rates [16], [17] and markers of inflammation (e.g., interleukin (IL)-6) of hospitalized COVID-19 patients [7], [18]. These findings suggest cardiac biomarkers could potentially be used to identify a subset of patients who are at a higher risk of poor clinical outcomes following COVID-19. Additionally, it is important to note that SARS-CoV-2 was found to be present in the interstitium of myocardial tissue, in addition to within cardiomyocytes.

Additionally, during the forum, Dr. Biykem Bozkurt from Baylor College of Medicine reemphasized that the prevalence of these cardiac abnormalities following SARS-CoV-2 infection have been alarming. One study found elevated levels of high-sensitivity cardiac troponin I (hs-cTn1) in 46% of non-survivors, in comparison to 1% in survivors [17]. Also, increased rates of myocardial infarction were also associated with COVID-19 [19]. This could be in part due to decreased availability of resources in the emergency setting or patients’ procrastination in obtaining treatment. A study by Puntmann, *et al.* showed 78% of patients who had recovered from COVID-19, regardless of their severity, had abnormal cardiac MRIs three months after recovery. Additionally, 71% of this cohort displayed biomarker abnormalities [20]. Over a two-week period in early 2020, Oxley, *et al.* identified five COVID-19-positive individuals younger than 50 years-old presenting with large-vessel ischemic stroke. The same research team identified, on average, 0.73 patients in an age-matched group over the previous year [21]. Additionally, elevated levels of D-dimer, commonly used to indicate blood clotting, were also observed to correlate to COVID-19 severity [3], [21], [22]. This evidence of clotting disorders was not limited to the cardiovascular system, and greatly impacted the neurovascular system as well [12].

Because COVID-19 can impact the cardiac system and could lead to heart damage, investigating cardiac recovery of individuals who require cardiovascular involvement during their daily activities, such as athletes, is particularly important. In a study conducted at The Ohio State University, 26 athletes who were recovering from COVID-19 underwent T2 late gadolinium enhancement (LGE) MRI. Their findings suggest there should be a long-term recovery phase with no exercise for at least two weeks regardless of the presence of symptoms or abnormal biomarkers [23], [24]. Additionally, the American Academy of Pediatrics suggested a recovery phase of 3-6 months for children who had recovered from severe cases of COVID-19 [25].

### C. COVID-19 Effects on the Brain and Neurological Diseases

SARS-CoV-2 can cause inflammation of the vascular system, which can potentially trigger neurological diseases and the subsequent cognitive and behavioral actions of the infected patient. Dr. Kristl Vonck from Ghent University, Belgium shared several studies that showed increased rates of encephalopathy and stroke in COVID-19 patients, unlike other coronavirus diseases (e.g., SARS, MERS). Although encephalitis requires a pathological diagnosis, physicians have begun to utilize other clinical markers such as imaging, focal EEG, and other behavioral or cognitive changes to decrease invasive and potentially infectious procedures typically required for diagnoses [26]. One study in France showed 84% of 58 ICU patients had neurological complications at discharge [27]. Additionally, a hallmark symptom of COVID-19 includes anosmia and ageusia, colloquially referred to as the loss of smell and taste without nasal congestion or rhinorrhea, respectively. Another European study observed 82% of COVID patients had gustatory dysfunction, 40% of which were able to recover in the early phase (1–2 weeks) post-infection (n = 417). However, the remaining 60% of these patients with anosmia and/or ageusia had prolonged duration of these symptoms long after viral shedding had ended [12], [28].

### D. COVID-19 Effects on Mental Health

In March 2020 a series of lockdowns were issued globally, limiting face-to-face interactions, and outdoor activities, which significantly impacted mental and physical wellbeing. As Dr. John Torous from Beth Israel Deaconess Medical Center mentioned during the forum, the presence of COVID-19 has been associated with an increase in the incidence of a first psychiatric diagnosis (typically anxiety or depression; Hazard Ratio ∼2) between 14–90 days. Similarly, a psychiatric diagnosis in 2019 was also associated with increased rate of COVID-19 diagnosis [29], [30]. This suggests COVID-19 and mental health influence each other. Longitudinal studies will need to be conducted to determine any physical changes within neurological tissue associated with the disease and associated impacts on mental health. Additionally, there has been an increased rate of mental illness among senior citizens, people of color, and lower socioeconomic status [29]. This could be further impacted by the continuing economic consequences to the pandemic as clearly indicated by Dr. Ross Zafonte from Harvard Medical School during the forum. To address these alarming rates of mental health diseases, clinicians have adapted to utilize telehealth through smartphones and apps. Using data collected from the smartphones, clinicians are able to predict relapses and provide care to patients virtually. Although these apps have their own inherent risks and limitations, they have been a way for clinicians to continue to monitor and obtain data from patients during the pandemic [31]. Additional innovative tools are further discussed in this position paper.

## IV. Grand Challenges in COVID-19 Screening/Tracing

One of the major challenges of this pandemic has been identifying and isolating infected individuals before spreading the virus to uninfected individuals. Early detection of the virus has most often been achieved using real time polymerase chain reaction (RT-PCR). Typically, individuals wait to be tested until they are exhibiting common symptoms (e.g., upper respiratory infection (URI), ageusia), or if they have had close contact with infected persons. Although this method of detection is reliable, the largest challenge of using this protocol is the availability of testing material, the 24–72 hours typical response time, and cost. Further, the existing testing protocol using RT-PCR often fails to identify patients before they have spread SARS-CoV-2 to others [32]. Other methods include antibody testing, where blood samples from individuals who believe they may have recovered from COVID-19 are used to detect antibodies to SARS-CoV-2. If antibodies are present, these results suggest the individual had recovered from the disease. Although these approaches are functional, there is still an unmet need to rapidly identify and isolate infected individuals before they spread the virus. To address this need, new innovative technologies that provide reliable and prompt testing and tracking are urgently required.

Initially developed tests for SARS-CoV-2 were limited and were in short supply. New technologies were desperately needed to address not only the high demand and volume, but also the accuracy and turnaround time for test results. Consequently, the National Institutes of Health (NIH) launched the Rapid Acceleration of Diagnostics (RADx) program [33]. This program brought together leaders and scientists from academia, business, and government as well as the Rockefeller and Bill and Melinda Gates Foundations. Dr. Nancy Gagliano from the National Institute of Biomedical Imaging and Bioengineering (NIBIB) presented their work to speed innovation for the development and implementation of these novel technologies to combat COVID-19. The early main barriers to testing largely included access and turnaround time. Typically, three days were required to get results from early PCR tests. This longer time frame was inadequate to isolate and prevent community spread of the disease. Additionally, the cost of early tests was high for both patients and insurance companies. Access to the early tests was also limited; often only those who had known exposure to the disease were allowed to be tested. Finally, once the market was able to catch up and access to these tests was easier, the ethical dilemmas of whom to test and how often to test asymptomatic individuals rose. Even today, industry and academia are still balancing free will vs. vaccine or testing requirements to return to work or school.

The RADx initiative was launched in April 2020 with an initial focus on diagnostics [34]. 136 applications were selected from ∼3000 submitted for the first round consideration which focused on viability and addressing the question, “does the science and structure of the company have the potential to produce a final marketable product”? The next round included a deep dive discussion with experts to work with the applicants to examine the scientific merit and results of each potential technology [35]. The third round consisted of a steering panel made of various experts to determine if the NIH should fund any of the 46 potential applications. 22 applications were then invited to the fourth round, in which the new technologies were rigorously tested for clinical accuracy, mock regulatory approval and scaling up production schemes. These 22 applications were very diverse; some were based on PCR-technologies, others were point-of-care (POC) diagnostics, others used antigen recognition and one applicant detected the virus in exhaled breath. As of November 2020, most of these companies were producing close to 6 million tests/day because of this program. Most recent estimates for diagnostic test production in 2022 include producing ∼300 million rapid tests per month in addition to the traditional PCR-based tests.

During the early stages of the pandemic, shortages of personal protective equipment (PPE) were not uncommon. Healthcare workers were reduced to reusing medical-grade isolation gowns throughout the entire shift, instead of changing for each patient. Additionally, single-use disposable surgical masks were no longer used for individual patients, but used for an entire shift, often >12 hours. Some hospital systems were only able to give their frontline workers one surgical mask every two weeks during the initial months of the pandemic. N95 masks to be used for COVID-19 positive patients during intubation or other aerosol-producing procedures were sometimes distributed once per quarter. As of January 2021, the US CDC guidelines continued to request civilians use cloth masks and reserve medical-grade ones for healthcare workers, in an effort to preserve medical-grade equipment for healthcare workers.

Not only was there a shortage of PPE, but also a need for more testing centers. During the forum, Dr. David Walt from Harvard Medical School shared his story of establishing the new Mass General Brigham Center for COVID innovation. This collaborative center first aimed to design and then produce enough PPE for local hospital systems. Additionally, Dr. Walt is the head of the “Diagnostic Accelerator” which aims to decrease the time needed for diagnostic test results. This physical facility acquires assays and other testing platforms and independently validates them using their team of full-time technicians and biobank samples. This is a way to remove product self-testing and induce bias towards statistically significant findings.

**Fig. 1.**
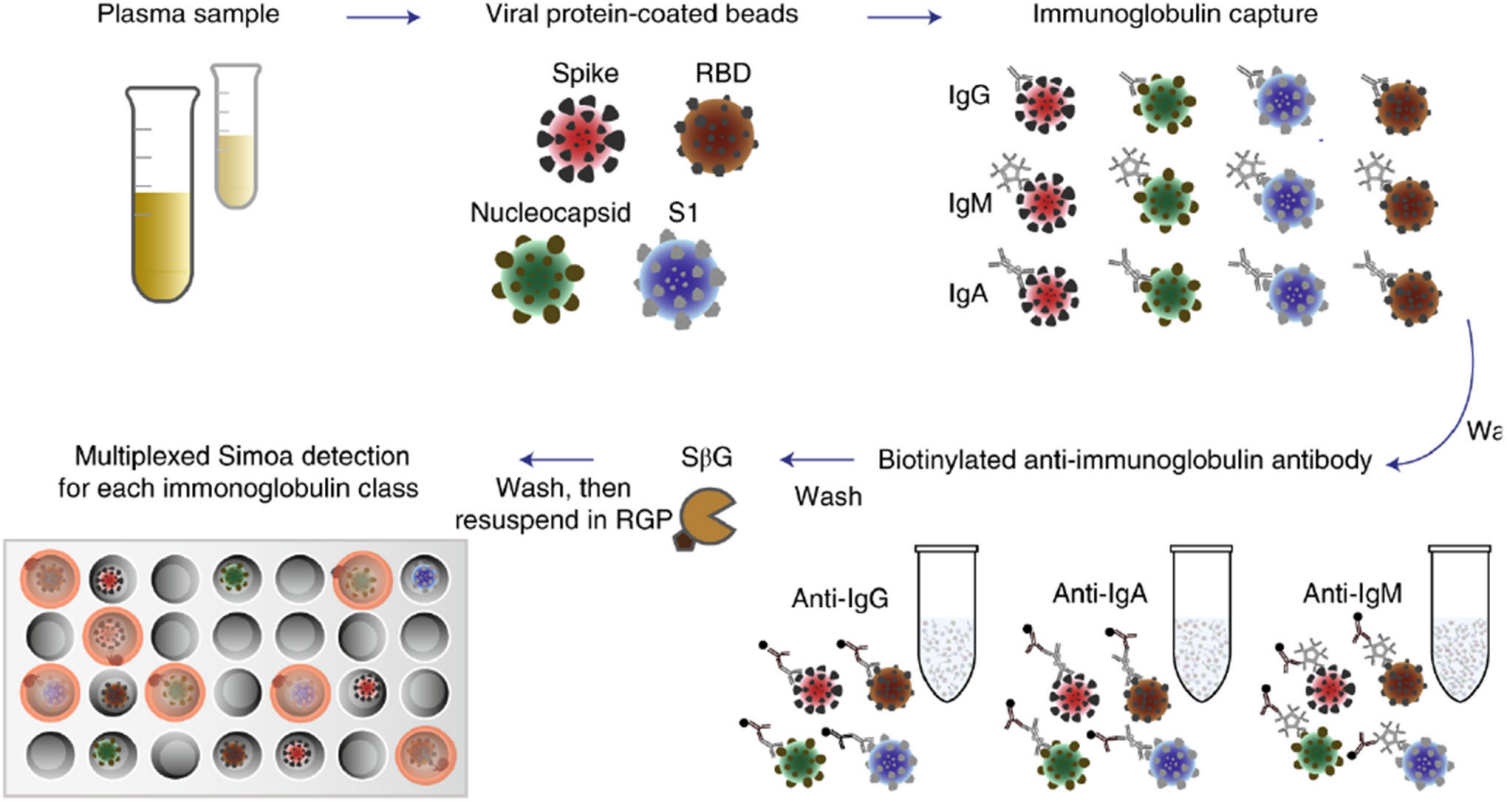
Plasma is incubated with four types of dye-encoded beads that are each coupled to one of four viral targets (spike, S1, RBD and nucleocapsid). IgG, IgA and IgM antibodies specific to the SARS-CoV-2 targets bind to the viral antigen-coated beads. After washing, beads are introduced to biotinylated anti-human immunoglobulin antibodies to label either IgG, IgM or IgA in the different reactions. After additional washes, the enzyme SβG is introduced. The beads are washed, resuspended in fluorogenic RGP and loaded into a 216000-microwell array for multicolor imaging. Courtesy of Norman *et al.* [36].

One promising new technology for rapid testing involves the use of ultra-sensitive protein detection using single molecule arrays (SiMoA). The SiMoA array captures the proteins on beads, similar to other multiplex assays, which are then isolated in individual wells. Each protein/antibody pair will have a specific, local florescent product that cannot travel to other wells. This digital response is a way to quickly validate the presence of a protein/antibody interaction. Dr. Walt's group looked for interactions between IgG, IgA and IgM antibodies with four viral protein-coated beads (spike, RBD, nucleocapsid, S1) as shown in Fig. 1 [36]. Using this novel array, they were able to find seroconversion using clinical samples collected on the day a patient presented to the emergency department. Serial samples from this patient revealed that the production of antibodies to SARS-CoV-2 proteins increased over time, as would be expected, for each of the four viral proteins. This preliminary data demonstrates this system can be used for rapid detection of the presence of proteins, and for personalized medicine applications.

With the increase in testing capabilities, Dr. Andrea Zanchettin, from Politecnico di Milano, has been developing a novel collaborative robotic platform to decrease the effort demanded for technicians by 62%, while increasing overall throughput (+66%). These collaborative robotic systems merge the flexibility of human manual labor with the efficiency of hard automation and could be an attractive and affordable addition to small hospitals to rapidly analyze clinical samples from patients with COVID-19.

Several new initiatives are incorporating new technologies with artificial intelligence (AI). Professor Achillefs Kapandis from the University of Oxford shared exciting new methods to rapidly detect the presence of the SARS-CoV-2 virus using single-particle imaging and deep learning algorithms. Using a custom microscope (i.e., Nanoimager), they image inside living cells, enabling quick detection of viral particles. Briefly, this method labels the viral particles with antibodies, aptamers, ssDNA, or fluorescent probes and then uses single-particle tracking and particle-size determination. If the probe is attached to a virus (∼125 nm), the very bright signal diffuses slowly, whereas free floating probes (2-5 nm) are dim and fast. This rapid detection method does not require purification or amplification of any gene and has already shown great promise with other viruses, including influenza or Respiratory Syncytial Viruses (RSV) [37]. Others have utilized convolutional neural networks (CNN) to determine a high or low probability of viral infection using images of different viruses [38]. These CNN-based methods are attractive because they can take minutes to analyze and detect viral particles, whereas other traditional methods including electron microscopy for viral detection, cell culture, and genome detection take hours, if not days to obtain results.

Additionally, there are several imaging modalities that can be utilized to rapidly screen patients for the presence of COVID-19. The quick turnaround time for image acquisition and analysis makes them potential candidates for use in rapid disease detection, especially in comparison to the days it can take to obtain PCR results. Dr. Jeffrey Kanne from the University of Wisconsin School of Medicine shared that imaging technologies were initially used to only track worsening conditions in COVID-19-positive patients, but have since been used to identify evidence of the disease even in asymptomatic individuals (later confirmed with standard PCR techniques). Current efforts are focused on finding the differences between other respiratory diseases with damaged or scarred lung epithelium (e.g., acute lung injury, influenza, autoimmune disease, vaping/smoking injury) and developing additional CT modalities to detect the presence of COVID-19 efficiently and accurately.

**Fig. 2.**
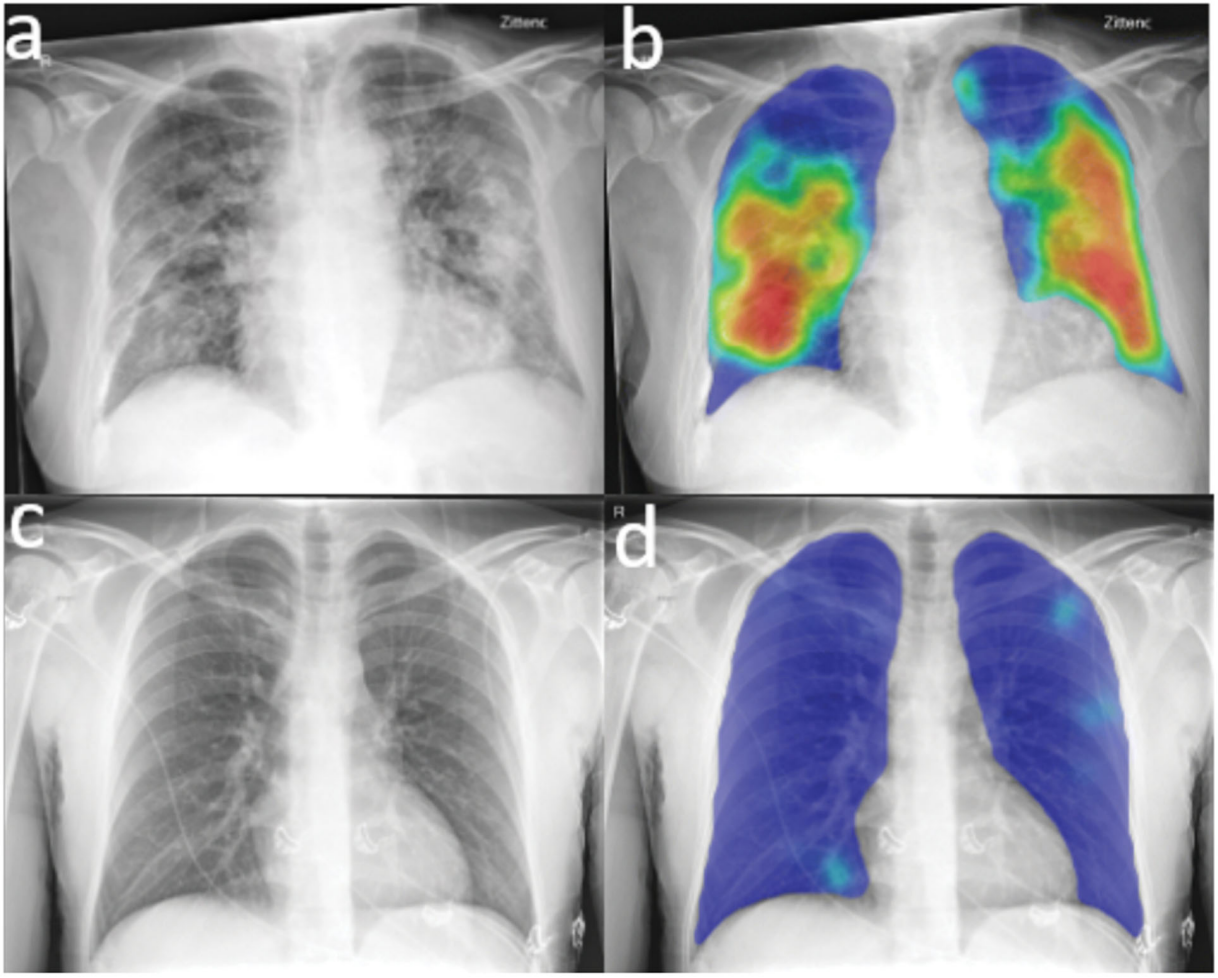
Top (a) and (b): Frontal chest radiograph images of a 74-year-old man with positive reverse transcription polymerase chain reaction (RT-PCR) test results for severe acute respiratory syndrome coronavirus 2 (SARS-CoV-2) viral infection. (b) Artificial intelligence (AI) system heat map overlaid on (a) shows pneumonia-related features. The AI system score for this subject is 99.8. Bottom (c) and (d): Images in a 30-year-old man with negative RT-PCR test results for SARS-CoV-2 viral infection. (c) Frontal chest radiograph. (d) AI system heat map overlaid on (c). The AI system score for this subject is 0.2. Courtesy of Murphy *et al.* [39].

Dr. Eva van Rikxoort from Thirona also shared how they are using imaging modalities, especially X-ray and CT to diagnose COVID. Imaging in combination with AI provides an opportunity to quickly identify positive cases during triage. Thirona has developed multiple products to quickly analyze X-ray (CAD4COVID-XRay) and CT (CAD4COVID-CT) images to produce an overall infection severity score [39], [40]. These deep learning networks were trained using cases collected worldwide. The output time for the X-ray algorithm is ∼30 seconds and produced a probability score for COVID-19 including a heat map of abnormal findings on the X-ray. As of November 2020, CAD4COVID-XRay has been used in 75 hospitals in 30 countries and has analyzed over 12000 images. Examples of X-ray images and the AI algorithm are shown in Fig. 2.

This AI system assessed CT severity scores per lobe which can be used to quantify lung damage during patient follow-up. Similarly the CT algorithm had been used in 47 hospitals in over 15 countries and has analyzed over 4300 CT scans. In the United States, it has been approved for FDA use during the length of the public health emergency. Lessmann, *et al.* were successfully able to combine CT with AI to detect COVID-19 and quantify severity comparable to that of radiologic observers [40]. This study in addition to the study by Murphy, *et al.* [39] demonstrate the potential of AI to support radiologists during busy periods (i.e., waves of SARS-CoV-2 infections).

Additionally, the COVID-19 pandemic has highlighted an urgent need to accelerate the digital transformation of healthcare systems. During Fluxergy CCO, Dr. Ali Tinazli's talk, we learned that diagnostics-related tests and procedures are 2% of total healthcare expenditures, but the outcome impacts 60% of critical decision-making. Recent advances in microfluidics and nanosensors have made, at home or mobile clinical sample, collection feasible, coupled with advanced sample stability/freshness/ to make digital health and personalized medicine relevant and feasible. Digital health now needs to be transferred to preventative medicine applications utilizing existing technologies, such as smart phones or electronic medical records. Fluxergy, Inc. has a low-cost, easy to use, and accurate multiplex platform with a CLIA wavier. Their platform can analyze various biomarkers, ranging from cells to molecules using real-time PCR, colorimetric detection/spectroscopy, electrical potentiometry, and imaging cytometry. This system also has multiple modalities and multiplex options, such that one can run a PCR and a serological test simultaneously. At the time of the forum in November 2020, they were still developing chips for the detection of SARS-CoV-2, RSV and influenza strains [41]. However, Fluxergy, Inc. announced they received the Conformité Européenne (CE)-in vitro diagnostic (IVD) marking for their one-hour COVID-19 RT-PCR Test on March 25th, 2021. This company will continue to use its existing infrastructure to tackle chronic disease monitoring, preventative care, and drug-resistant “superbugs.”

## V. Grand Challenges in COVID-19 Prevention and Treatment

Vaccines are a way to create an immune population without risking high rates of death and infection to reach the same level of immunity. Additionally, vaccinated individuals within an immune population are expected to have lower rates of infection and severity of disease in comparison to unvaccinated populations. During pandemics, scientists must be quick and precise to accurately develop vaccines to save lives. Academic partnerships are key accelerators of vaccine development because they are a source of innovation, specialized knowledge and can cut costs and mitigate risks while rapidly transitioning from basic research to late-stage development of vaccine technology. During the SARS-CoV-2 pandemic, scientists worldwide have used existing technology obtained from other SARS-like viruses (e.g., MERS, SARS) to rapidly develop safe and effective vaccines. Several of the frontrunners in vaccine development include using a structure-based vaccine design, in which the vaccine aims to allow the body to produce antibodies which can then interfere with the SARS-CoV-2 spike protein - ACE2 receptor interactions, preventing viral DNA transfection.

At the time of the Public Forum, there were 47 vaccine candidates worldwide in clinical evaluation, using nucleic acid technology, whole-inactivated viruses, recombinant vectors, recombinant proteins, recombinant or chimeric viruses, virus-like particles or peptides. Additionally, there were 155 vaccine candidates in pre-clinical evaluation, using nanoparticles, live-attenuated viruses, recombinant vectors, recombinant proteins, recombinant or chimeric viruses [42]. Dr. Hanneke Schuitemaker, Global Head of Viral Vaccine Discovery and Translational Medicine, and Disease Area Stronghold Leader for Viral Vaccines at Janssen Vaccines and Prevention, opened the fourth and final day of the Public Forum. She shared exciting data regarding their one-dose COVID-19 vaccine. This vaccine utilizes the same technology which was used to develop other vaccines (e.g., Ebola, Zika, etc.), which uses an inactivated common cold virus Adeno 26 to encode a stabilized variant of the SARS-CoV-2 Spike (S) protein. Unlike other high-profile vaccine candidates (e.g., Pfizer, Moderna, Oxford/AstraZeneca), the Janssen vaccine does not utilize mRNA technology, and is stable at 4°C [43], [44]. This vaccine was approved for Emergency Use Authorization by the U.S. Food and Drug Administration (FDA) on February 27, 2021 [45].

Dr. Maria Elena Bottazzi is the Co-Director of Texas Children's Hospital Center for Vaccine Development at Baylor College of Medicine. She emphasized the importance of global access to vaccines and shared how their center works to ensure the scalability, quality and functionality of vaccines for global recipients. Using technology previously developed to address the SARS pandemic over a decade ago [46], [47], they have developed a new vaccine candidate in approximately 5 months, in partnership with Merck MilliporeSigma. Recently, they developed a variant of the recombinant receptor-binding domain of the SARS-CoV-2 spike protein in yeast which also binds to the ACE-2 receptor [48]. Yeast are less expensive than mammalian cell lines, which makes this vaccine candidate potentially able to expand vaccine access, thereby bridging the gap between low- and middle-income countries. This vaccine candidate was licensed to Biological E, Ltd. based in India in July 2020, where a Phase 1/2 trial just began. Biological E has vast experience with yeast vaccines and has already scaled up to produce 1.2 billion doses/year.

**Fig. 3.**
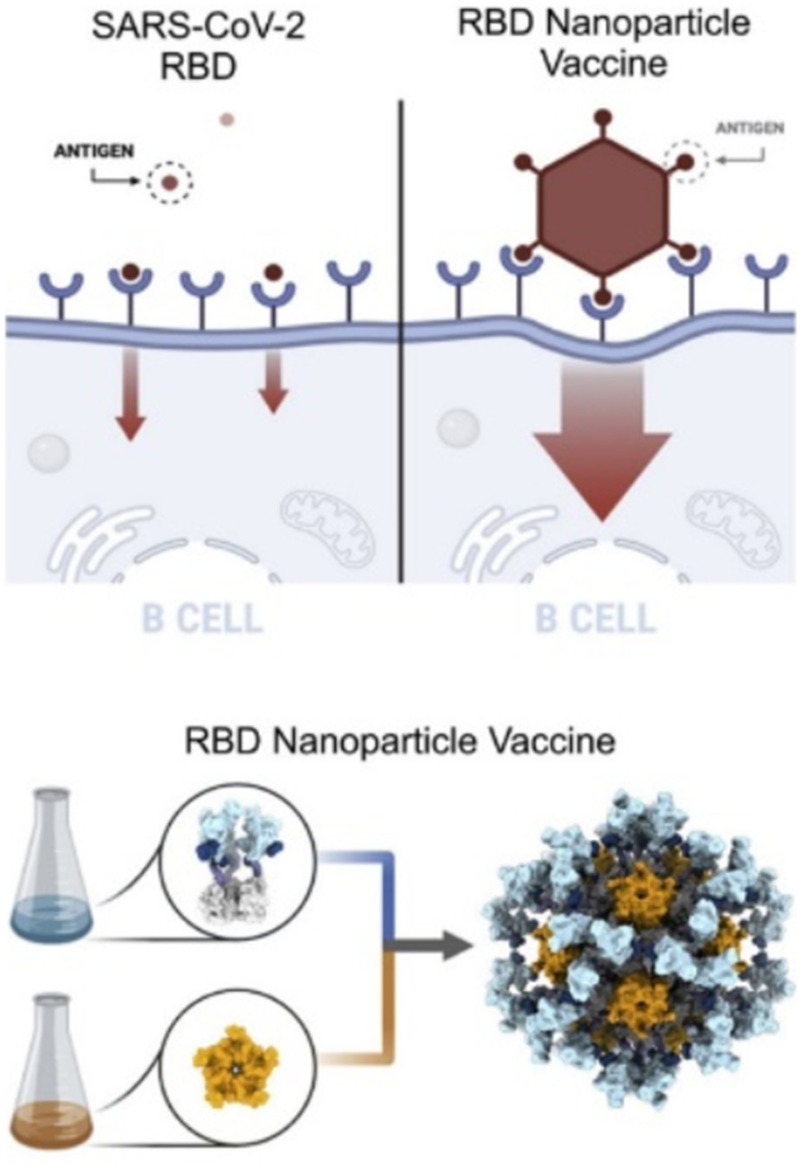
Overview of self-assembled nanoparticle to mimic SARS-CoV-2 infection. Courtesy of Walls *et al.* [11].

Additionally, Dr. Neil King from the University of Washington described his new technology to create self-assembled nanoparticles using proteins but no viral particles. Specifically, his nanoparticles are designed using symmetry and non-covalent protein interactions, allowing for customizable structures to fine tune porosity and overall nanoparticle size. They successfully designed and created self-assembled nanoparticles for respiratory syncytial virus (RSV), influenza, and more recently, the SARS-CoV-2 virus [11]. This nanoparticle contains the receptor-binding domain of the SARS-CoV-2 spike protein and is stable up to 28 days when stored at −80°C or room temperature (22–27°C). Murine models showed a promising immune response after the second dose. The nanoparticle currently is being manufactured for exploration of clinical trials. A summary of his method is shown in Fig. 3.

There have been several proposed treatments for COVID-19, including antimalarial drugs, antiviral agents, immunomodulatory, glucocorticoids, and convalescent plasma [9]. Dr. Barney S. Graham, Deputy Director for the Vaccine Research Center at the National Institute of Allergy and Infectious Diseases (NIAID) at the NIH shared how this institute is utilizing several technologies to develop COVID-19 treatments. Specifically, he shared how an anti-COVID 19 antibody therapeutic, bamlanivimab, was developed. Using blood processed at NIH in February of 2020, Chen, *et al.* discovered COVID-19 β-cells which were cloned at AbCellera, Inc. This was used to create antibodies and start a Phase 1 clinical trial; the antibodies were screened at NIH and manufactured by Eli Lilly. Chen *et al.* reported results from the stage 2 trial. This antibody treatment was used to treat mild or moderate cases of COVID-19 and showed a rapid decrease in viral load and decreased incidence rates of hospitalization (6% untreated vs. 1.6% treated). This represented a 72% decrease in disease progression [9]. Commercially recognized as bamlanivimab, this monoclonal antibody treatment was announced for EUA by the FDA on November 9, 2020. After the EUA was revoked on April 16, 2021 [49], the FDA reissued an EUA for bamlanivimab when administered with etesevimab together, on September 16, 2021 [50].

**Fig. 4.**
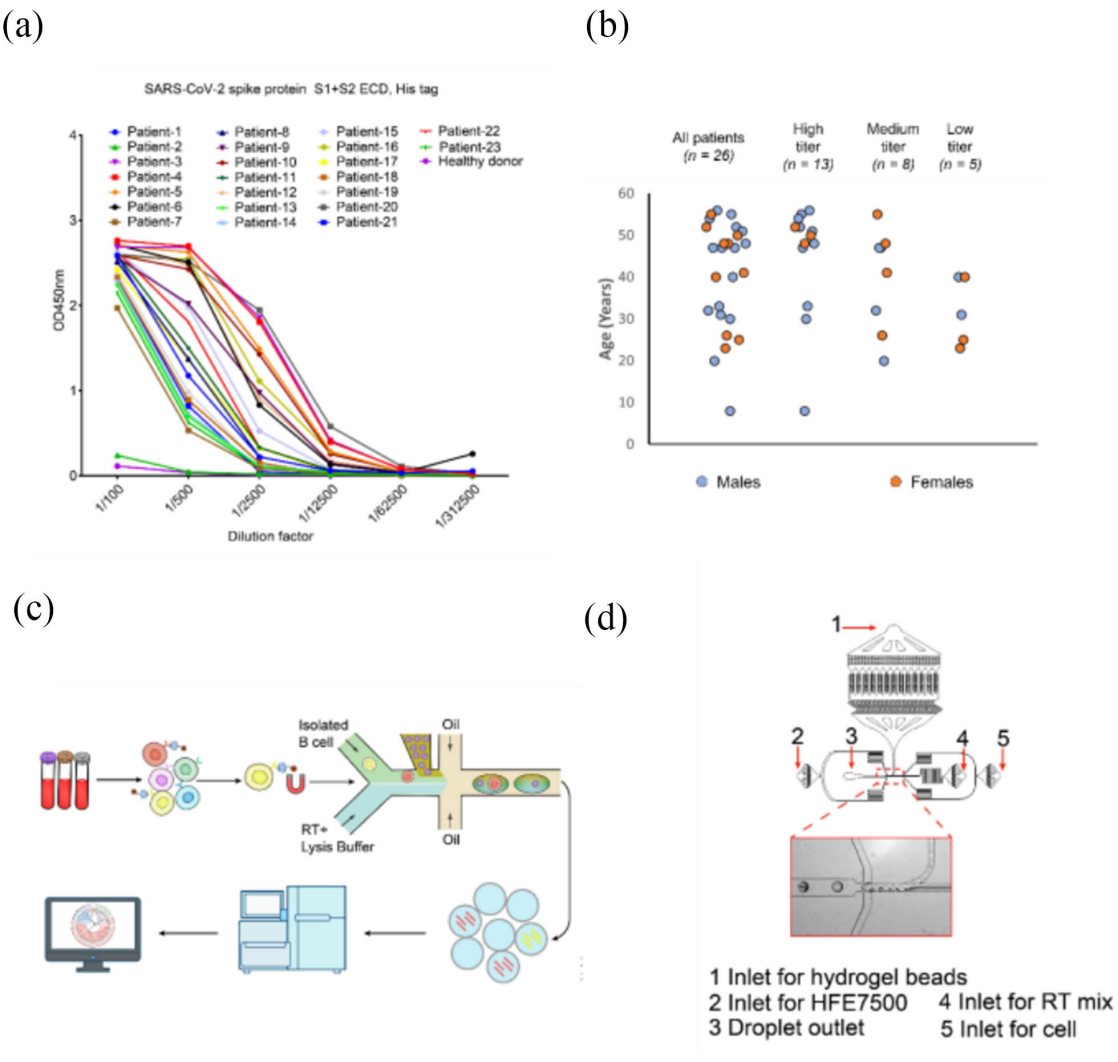
Analyses of antibody responses to SARS-CoV-2 proteins and antibody identification from convalescent patients using single B cell sequencing. (a) Sera from 23 convalescent patients and one healthy donor were analyzed for their binding abilities to the SARS-CoV-2 spike protein using ELISA. Samples at different dilutions were tested in duplicates and mean is shown. (b) Classification of patient samples into high (>2500), medium (500–2500), and low (<500) titer categories. (c) Schematic diagram of the antibody identification from convalescent patients using single B cell sequencing. SARS-CoV-2 S protein binding B cells were isolated from PBMC of convalescent patients with magnetic beads that conjugated with biotinylated S protein as probes. The isolated cells were individually co-compartmentalized in droplets along with lysis buffer, reverse transcriptase and one hydrogel bead. Each hydrogel bead carried VH and VL specific oligos tagged with a unique barcode. The resulting cDNAs from one cell carried an identical barcode. The barcoded cDNAs were sequenced to identify cognate heavy and light chain pairs. (d) The design of the microfluidics chip for co-compartmentalization of single cells and single hydrogel bead in droplets. Courtesy of Guo *et al.* [10].

Dr. Liang Schweizer, Co-Founder, President and CEO of HiFiBio Therapeutics shared her company's exciting new treatment for COVID-19. They recently developed a monoclonal antibody treatment by isolating and identifying neutralizing antibodies binding to SARS-CoV-2 Spike protein from COVID-19 convalescent patients (Fig. 4). They found that one antibody, P4A1, shows potent neutralizing activities and binds and covers the majority of the binding site of ACE2. Additionally, they engineered P4A1 (P4A1–2A) and determined efficacy using rhesus macaques, demonstrating potent anti-viral efficacy following a single injection [10]. We believe these treatment options provide complementary methods to vaccine approaches to protect vulnerable populations and control the COVID-19 pandemic.

## VI. Perspectives on Addressing Ongoing and Future Challenges

This paper summarizes the IEEE EMBS Public Forum on COVID-19. During the pandemic, scientists, clinicians, and engineers have been able to join forces and create novel diagnostic tools, as well as therapeutic treatments to combat the novel COVID-19 pandemic. Subsequently, hospitals and companies were able to utilize these new technologies. Communities also responded to the challenges presented by the COVID-19 pandemic by transitioning to remote operations; schools transitioned to virtual learning, offices adopted work from home strategies, and mass gatherings werediscouraged in 2020 and 2021 [51]. The joint effort of government officials, public health experts, and healthcare industry scientists was indeed, remarkable and can be attributed to saving many lives. However, we believe the lack of a global collaborative strategy hindered community attempts to “flatten the curve,” slow the spread of the virus, prevent mortality, and lessen the economic burden of this disease, especially within communities of color and those who are economically disadvantaged worldwide.

As of January 15^th^, 2022, 59.9% of the worldwide population has received at least one dose of a COVID-19 vaccine [52]; 62.5% of the United States [52] and 69.1% of Europe [53] are fully vaccinated. Some countries and regions, including the European Union (EU), are creating digital vaccine certificates to coordinate travel measures and ensure free movement and safety of citizens, while decreasing transmission of the SARS-CoV-2 virus. The EU Digital COVID Certificate is proof the individuals has received up-to-date vaccinates, recently recovered from an infection, or has a negative COVID test. This free certificate is available digitally or on paper, is valid in all EU countries, and can be provided in the national language as well as English. Additionally, 33 non-EU countries have adopted the EU Digital COVID certificate [54]. Recently, countries are allowing vaccinations of children ages 5–18, which will lessen the social, mental, and educational burden this virus has had on children. Although access to one pediatric vaccine for 5–18 year-olds is encouraging, there is an urgent need to hasten the development of a safe and effective COVID-19 vaccine for younger children. Additional consideration of annual vaccinations for COVID-19, similar to the seasonal influenza vaccine should also be addressed. We strongly believe that until the pandemic is completely and globally contained, citizens should be encouraged to wear masks in public [55], and should follow social distancing guidelines.

Furthermore, developing additional therapeutic treatment options should also include the development of antiviral medications for those with COVID-19. Although the vaccination of frontline healthcare workers and government officials has been highly encouraging as a first step to build herd immunity against SARS-CoV-2, there is an unmet and urgent need for a stronger global partnership, healthcare reform, and more equitable vaccine and data distribution to combat the current pandemic and prevent future ones. This lack of global partnership and vaccination-equity has directly led to the rise of additional variants, including delta and omicron [4], [5], [56]. Therefore, a comprehensive and global vaccination plan is urgently needed to prevent additional variants, which could challenge current vaccines, and contain the spread of the SARS-CoV-2 virus.
